# From Embryogenesis to Senescence: The Role of Mammary Gland Physiology in Breast Cancer Risk

**DOI:** 10.3390/cancers17050787

**Published:** 2025-02-25

**Authors:** Jaida C. Lue, Derek C. Radisky

**Affiliations:** 1 Graduate School of Biomedical Sciences, Mayo Clinic, Jacksonville, FL 32224, USA; 2 Department of Cancer Biology, Mayo Clinic, Jacksonville, FL 32224, USA

**Keywords:** mammary gland development, breast cancer risk, embryonic development, postlactational involution, age-related lobular involution (ARLI), cellular senescence, senescence-associated secretory phenotype (SASP), tumor microenvironment (TME), extracellular matrix (ECM), immune modulation, postpartum breast cancer (PPBC)

## Abstract

The mammary gland undergoes significant changes throughout a woman’s life; from embryonic development to transformations after breastfeeding and during aging. These processes, while essential for normal breast physiology, can increase breast cancer risk when disrupted. This review explores three critical stages: embryonic development; postlactational involution; and age-related lobular involution (ARLI). We highlight key signaling pathways—Wnt, FGF, SHH, Notch, EGFR, and BMP—that guide embryonic development and discuss how their dysregulation can contribute to abnormal growth. For postlactational involution, we examine the two-phase process of cell death and tissue remodeling, showing how disruptions during this period, particularly postpartum, may foster a tumor-promoting environment. We also delve into ARLI and the role of cellular senescence in the aging mammary gland, focusing on the senescence-associated secretory phenotype (SASP) and its impact on inflammation and tissue remodeling. Understanding these processes provides new opportunities for breast cancer prevention and treatment strategies

## Simple Summary

Breast cancer is one of the most common cancers in women, and understanding its risk factors is critical for improving prevention and treatment. The mammary gland changes significantly throughout life, from its early development in the womb to transformations during pregnancy, breastfeeding, and aging. These natural processes, while essential, can sometimes become disrupted and increase the risk of breast cancer. This review explores how the mammary gland develops, how it remodels after breastfeeding, and how it ages, highlighting the cellular and molecular changes that may lead to cancer. By studying these processes, scientists can identify key factors that contribute to breast cancer risk and develop new strategies to prevent or treat the disease. This research aims to deepen our understanding of breast cancer and inspire innovative approaches to protect women’s health.

## 1. Introduction

Breast cancer remains one of the most prevalent malignancies among women worldwide, contributing significantly to illness and death. Its causes are multifactorial, arising from a complex interplay of genetic, hormonal, environmental, and physiological factors. Among these, the physiological processes governing mammary gland development and remodeling play a pivotal role in shaping breast cancer risk throughout a woman’s life.

The mammary gland is a dynamic organ, undergoing continuous remodeling in response to life stages such as embryonic development, pregnancy, lactation, and aging. During embryogenesis, key signaling pathways—including Wnt, fibroblast growth factors (FGFs), Sonic hedgehog (SHH), Notch, epidermal growth factor receptor (EGFR), and bone morphogenetic proteins (BMPs)—guide the formation of the mammary gland’s foundational structure. Disruptions in these tightly regulated pathways can predispose individuals to breast cancer later in life by influencing cellular proliferation, differentiation, and survival.

Following pregnancy and the cessation of lactation, the mammary gland undergoes postlactational involution, a normal process of tissue remodeling that restores the gland to its pre-pregnancy state. This transformation involves programmed cell death and extracellular matrix (ECM) remodeling. However, aberrations in this process can create a tumor-promoting microenvironment. Postpartum breast cancer (PPBC), defined as breast cancer diagnosed within a decade of childbirth, is particularly aggressive and often has a higher risk of metastasis.

In later life, the mammary gland experiences age-related lobular involution (ARLI), a gradual regression of glandular structures accompanied by the accumulation of senescent cells. While ARLI and cellular senescence help limit the number of potentially malignant cells, their dysregulation can lead to chronic inflammation, ECM alterations, and immune dysfunction—all of which contribute to an increased risk of breast cancer. Senescent cells release pro-inflammatory and tissue-remodeling factors, collectively known as the senescence-associated secretory phenotype (SASP), which further amplify this risk by fostering a pro-tumorigenic environment.

This review examines how mammary gland development and involution influence breast cancer risk, with a focus on three critical stages: embryonic development, postlactational involution, and ARLI. By exploring the molecular and cellular mechanisms underlying these processes, we aim to highlight potential therapeutic targets for breast cancer prevention and treatment strategies.

## 2. Early Mammary Gland Development

Embryonic mammary gland development is a tightly regulated process that establishes the foundation for a functional mammary gland in later life. Disruptions in these processes can lead to abnormal growth patterns and contribute to the initiation and progression of breast cancer. Although much of our knowledge comes from mouse models, which are extensively studied, the underlying molecular mechanisms are largely conserved across mammals, including humans.

### 2.1. Mammary Line Formation

**Figure 1:**
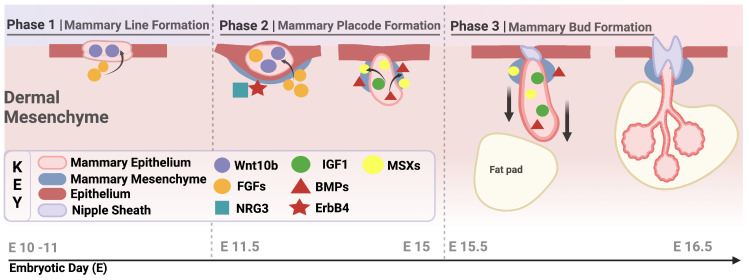
The key stages of embryonic mammary gland development. This schematic illustrates the sequential phases of mammary gland development during embryogenesis in mice, highlighting critical cellular components, molecular signals, and morphological changes. Phase 1: Mammary Line Formation (E10–E11): The mammary ridge, a thickened band of ectodermal tissue, emerges along the ventral surface, driven by epithelial–mesenchymal interactions and signaling pathways, such as Wnt10b and FGFs. Phase 2: Mammary Placode Formation (E11.5–E15): Localized thickenings, termed mammary placodes, develop along the mammary ridge. These structures involve Wnt10b, FGFs, NRG3, and ErbB4 signaling, preparing for bud invagination. Phase 3: Mammary Bud Formation (E15–E16.5): Mammary buds form and invade the underlying mesenchyme, guided by ECM remodeling and molecular signals. The buds extend into the fat pad, establishing the rudimentary ductal system. Key: The diagram identifies major signaling pathways (e.g., Wnt10b, FGFs, BMPs), cell types (e.g., mammary epithelium, mesenchyme), and structural features (e.g., nipple sheath, fat pad) involved at each stage. The timeline (embryonic day) is aligned with mouse development.

The first stage of embryonic mammary gland development begins with the formation of the mammary ridge, or milk line, at embryonic day 10.5 (E10.5) in mice (Figure 1) [1]. This ridge is a thickened band of ectodermal tissue that extends bilaterally along the ventral surface of the embryo from the forelimb to the hindlimb. Its development depends on interactions between epithelial and mesenchymal cells [2,3,4], mediated by multiple signaling molecules and transcription factors. Key players include the Msx homeobox genes and bone morphogenetic proteins (BMPs). Msx-1 and Msx-2, members of the homeobox gene family, are crucial for tissue patterning and organogenesis [5,6]. BMPs, particularly BMP-2 and BMP-4, regulate cell proliferation, differentiation, and apoptosis. The coordinated expression of Msx genes and BMPs is critical for initiating the epithelial–mesenchymal interactions that drive mammary ridge formation [6,7,8].

Another pivotal pathway is Wnt signaling, which specifies cells that will form the mammary line and later become restricted to mammary placodes [9]. The expression of Wnt10b is one of the earliest events detectable in mammary lines. Additionally, fibroblast growth factors (FGFs) and their receptors contribute significantly to this stage. For example, Fgfr2b expression is observed at E11.5 and E15.5, while Fgf7 is expressed in the mammary mesenchyme at E12.5 [10].

#### 2.1.1. Mammary Placode Formation

Between E10 and E11, five pairs of mammary placodes develop along the mammary ridge [11]. These placodes, localized thickenings of ectoderm, give rise to mammary buds and eventually invaginate into the mesenchyme, forming the nipple and ductal network [2,12,13]. Placode development is driven by epithelial–mesenchymal interactions and regulated by multiple signaling pathways, including the Sonic hedgehog (SHH) pathway, which mediates these interactions [2,14]. FGF signaling also remains essential during this stage. FGF10 and its receptor FGFR2b are critical, as knockout models lacking these factors show disruptions in four out of the five mammary placodes [15]. Other FGFs, such as FGF4, FGF8, and FGF17, and receptors like FGFR1, are also involved [10]. Neuregulin-3 (NRG3), part of the epidermal growth factor (EGF) family, and its receptor ErbB4, play indispensable roles in amplifying and expediting Wnt signaling, facilitating communication between the ectoderm and mesoderm [16,17,18]. These interconnected pathways ensure proper placode formation and subsequent mammary gland development.

#### 2.1.2. Bud Formation and Terminal End Buds (TEBs)

Mammary buds form in the third stage of development, ultimately giving rise to the ductal system [13]. Bud formation involves cell proliferation, migration, and differentiation, orchestrated by genetic and environmental factors. Terminal end buds (TEBs)—highly proliferative structures—drive ductal elongation and branching during postnatal development but have their foundation established during embryogenesis [19]. Although embryonic development is largely hormone-independent, estrogen and growth hormone synergistically influence TEB formation [20]. Growth hormone acts via paracrine insulin-like growth factor-1 (IGF-1) signaling, promoting cell proliferation and survival [21]. Additionally, the extracellular matrix (ECM) provides structural and biochemical support during bud formation [22]. ECM components, including collagen and fibronectin, interact with cell surface receptors like integrins to modulate cell behavior [23]. Matrix metalloproteinases (MMPs) play a key role in degrading ECM components, facilitating the invasion of mammary buds into the fat pad [11]. At E14, the mammary mesenchyme experiences condensation, resulting in a few layers of fibroblast-rich cells. The maturation process of the mesenchyme is influenced by the surrounding epithelium and can be distinguished from the formation of the fat pad precursor mesenchyme, which develops from deeper subcutaneous mesenchymal cells [11]. This coordinated degradation and remodeling of the ECM enables ductal branching morphogenesis, resulting in a rudimentary ductal tree composed of a primary duct and 15–20 secondary branches, laying the groundwork for future development [12,13].

### 2.2. Genetic and Environmental Factors Influence Mammary Gland Development

Genetic and environmental factors significantly influence early mammary gland development and breast cancer susceptibility. Approximately 5–10% of breast cancer cases result from hereditary factors [24]. BRCA1, BRCA2, and TP53 mutations are the most well-known contributors, significantly increasing breast cancer risk [25]. BRCA1 and BRCA2 mutations impair DNA repair mechanisms, leading to genomic instability [26]. Mutation carriers face a cumulative breast cancer risk by age 70 of approximately 65% for BRCA1 and 45% for BRCA2 [27]. TP53 encodes the p53 protein, a crucial regulator of the cell cycle and apoptosis [28]. Mutations in TP53, a critical regulator of the cell cycle and apoptosis, underlie Li–Fraumeni syndrome, which includes early-onset breast cancer [29,30]. However, individuals with BRCA1, BRCA2, and TP53 mutations do not have equal likelihood of developing cancer. Risk varies due to factors such as allelic heterogeneity, modifier genes, and environmental cofactors.

Environmental factors, responsible for 90–95% of breast cancer cases [31], also impact development. Early-life exposures to ionizing radiation, hormonal therapy, obesity, and dietary habits have long-term effects [32,33,34]. Ionizing radiation exposure, especially during puberty or early adulthood, increases breast cancer risk by causing DNA damage and genomic instability [35,36]. Elevated hormone levels, including estradiol and testosterone, are associated with increased risk [37,38,39,40].

Endocrine disruptors, such as Bisphenol A (BPA) and phthalates, interfere with hormone signaling during critical developmental periods. These compounds, found in daily-use items, can cause epigenetic modifications linked to breast cancer risk [41,42]. Additionally, obesity and adiposity during development alter hormone levels, reducing estrogen responsiveness and inhibiting ductal growth [43,44]. Reproductive history also influences risk; early pregnancy (before age 35) is associated with an initial increased breast cancer risk for approximately 10 years post-pregnancy, followed by a long-term protective effect. This long-term risk reduction is likely due to the differentiation of mammary epithelial cells during pregnancy [45].

## 3. Implications for Breast Cancer Initiation and Progression

### 3.1. Dysregulation of Developmental Pathways

The proper regulation of molecular pathways and processes involved in early mammary gland development is critical for establishing functional mammary tissue in adulthood. The dysregulation of these pathways can contribute to the initiation and progression of breast cancer. The Wnt signaling pathway, essential for mammary line specification and placode formation, is frequently implicated in breast cancer when abnormally activated [9,46]. The overexpression of Wnt ligands, such as Wnt1, Wnt3, and Wnt10b, has been associated with specific breast cancer subtypes [9]. Dysregulated Wnt signaling promotes increased cell proliferation and survival, contributing to tumor growth and metastasis.

Similarly, FGF signaling, critical during placode development, plays a role in breast cancer progression when deregulated [15]. The overexpression of FGF receptors, such as FGFR1 and FGFR2, enhances angiogenesis, promoting tumor growth and metastasis [15,47]. Mutations and amplifications in FGF receptors are frequently observed in various breast cancer subtypes, underscoring the importance of maintaining tightly regulated FGF signaling for normal cellular functions. The Sonic hedgehog (SHH) pathway, which governs placode development through epithelial–mesenchymal interactions, is also implicated in breast cancer progression. The dysregulation of SHH signaling has been explored as a potential therapeutic target in breast cancer. The abnormal overexpression of SHH or its receptor, PTCH1, in transgenic mouse embryos causes severe mammary bud malformations, including the complete absence of mammary buds [48]. Notch signaling, essential for embryonic mammary development, tissue homeostasis, and organogenesis, is similarly involved in breast cancer. Its dysregulation has been linked to endocrine resistance and disease progression [49,50,51]. The epidermal growth factor receptor (EGFR) pathway, which regulates cell growth, differentiation, and morphogenesis, becomes oncogenic when disrupted. The dysregulation of EGFR signaling leads to uncontrolled cell proliferation and invasiveness in breast cancer [52,53]. The overexpression of EGFR is frequently associated with poor prognosis and aggressive tumor behavior. Lastly, bone morphogenetic protein (BMP) signaling, which interacts with parathyroid hormone-related protein (PTHrP) during ductal morphogenesis, has been implicated in cancer when altered. Aberrant BMP expression affects cell differentiation and proliferation, and BMPs are known to influence the behavior of cancer stem cells. Targeting BMP signaling has been proposed as a therapeutic strategy for breast cancer [54,55,56].

### 3.2. Tumor Microenvironment and Breast Cancer Progression

The breast tumor microenvironment (TME) plays a critical role in supporting tumor growth and invasion [57]. Components of the TME—including immune cells, stromal cells, and the extracellular matrix (ECM)—interact with tumor cells to promote angiogenesis, immune evasion, and metastasis [57]. Changes in ECM composition and stiffness can alter tumor cell behavior, facilitating cancer progression. Additionally, tumors recruit immunosuppressive cells, such as regulatory T cells and myeloid-derived suppressor cells, (MDSCs) to evade immune surveillance. Angiogenesis, driven by factors like vascular endothelial growth factor (VEGF), provides tumors with nutrients and oxygen, further supporting their expansion.

### 3.3. Therapeutic Implications

Disruptions in normal developmental interactions between mammary cells and their microenvironment can predispose tissue to malignant transformation. Understanding these interactions is essential for developing targeted therapies and improving patient outcomes. For instance, therapies targeting the ECM components, angiogenesis, or immune checkpoints are being explored to disrupt the supportive TME and inhibit tumor growth.

In summary, early mammary gland development involves a complex interplay of signaling pathways and cellular interactions. The dysregulation of pathways, such as Wnt, FGF, SHH, Notch, EGFR, and BMP, can predispose individuals to breast cancer by promoting abnormal cell proliferation, survival, and tissue remodeling. A deeper understanding of these pathways offers potential for early detection, prevention, and targeted therapeutic strategies. Continued research is essential to unravel the link between normal and pathological mechanisms, ultimately translating this knowledge into improved clinical outcomes.

## 4. Postlactational Involution

Postlactational involution is a highly orchestrated process through which the mammary gland remodels itself after breastfeeding cessation, returning to a non-lactating state. This transformation involves extensive programmed cell death (PCD) and structural remodeling, replacing secretory epithelium with adipocytes [58]. While rodent models, particularly mice, have provided much of our mechanistic understanding, in which the process occurs rapidly within days, human postlactational involution is more gradual, requiring approximately 18 months postpartum for complete regression of pregnancy-associated differentiated lobules [59]. These differences reflect distinct reproductive patterns across species [60,61]. Understanding the molecular mechanisms underlying this process and translating findings from animal models to human biology is critical, as disruptions in postlactational involution are associated with increased breast cancer risk during the postpartum period.

### 4.1. First Stage: Lysosomal-Mediated Cell Death (LCD)

The initial phase of postlactational involution is driven by lysosomal-mediated cell death (LCD), which is distinct from classical apoptosis that relies on executioner caspases. LCD involves the detachment of epithelial cells from alveolar structures and their subsequent shedding into the lumen. Immune responses play a complementary role, with neutrophils initiating the response, followed by macrophages and lymphocytes, which orchestrate cell clearance and tissue remodeling [62].

The cessation of breastfeeding, or weaning, leads to milk accumulation in the alveolar lumen and a drop in circulating prolactin levels. This reduction induces the localized production of signaling mediators, including interleukin-6 (IL-6), leukemia inhibitory factor (LIF), and oncostatin M (OSM) [63,64]. The withdrawal of prolactin-induced STAT5 signaling results in a surge in IL-6 and LIF levels, activating the gp130 receptor and initiating LIF-induced STAT3 signaling, a pivotal event in this stage [65]. STAT3 signaling depends on Janus Kinase 1 (JAK1); in its absence, apoptosis is stalled, delaying involution [64,65].

During involution, STAT3 activation drives mammary epithelial cells to transition from a milk-producing secretory state to a phagocytic state while directly suppressing the PI3K-AKT survival pathway through reduced AKT phosphorylation [66,67]. STAT3 upregulates pro-apoptotic genes, such as Bcl-2-associated X protein (BAX), by altering mitochondrial membrane permeability. This dual action—the promotion of phagocytosis and the suppression of survival signals—enables epithelial cells to engulf dying cells and milk components, particularly milk fat globules (MFGs). The process is enhanced by the significant upregulation of milk fat globule EGF factor 8 (MFGE8), which acts as a bridging molecule by binding to apoptotic epithelial cells and facilitating their clearance by both neighboring epithelial cells and macrophages [68,69]. Simultaneously, STAT3 upregulates pro-apoptotic genes, particularly BAX, which increases mitochondrial membrane permeability and promotes cytochrome C release to trigger apoptosis. Together, these coordinated mechanisms—decreased AKT survival signaling, enhanced phagocytic capacity, and BAX-mediated apoptosis—ensure efficient cell death and clearance during the involution process.

This orchestrated cell death program is further enhanced through lysosomal-mediated cell death (LCD). A key feature of LCD is lysosomal membrane permeabilization (LMP), induced by tumor necrosis factor-alpha (TNF-α), which releases cathepsin proteases into the cytosol [70,71,72]. STAT3 upregulates cathepsins B and L while downregulating their inhibitor, Spi2A, ensuring efficient cell death [73]. Concurrently, transforming growth factor-beta 3 (TGFB3) facilitates cell junction reorganization and aids in clearing phagocytosed cells [74]. Remarkably, this process is reversible in mouse models if lactation resumes within 48 h, demonstrating the dynamic plasticity of the mammary gland [58].

### 4.2. Second Stage: Mitochondrial-Mediated Apoptosis and Tissue Remodeling

Approximately 48 h post-weaning in mice, the mammary gland transitions into the irreversible second phase, characterized by significant remodeling, including degradation of the basement membrane, alveolar collapse, and adipocyte repopulation [58]. Central to this phase is the activity of proteases, such as matrix metalloproteinases (MMPs), including MMP2, MMP3, and MMP9, and urokinase-type plasminogen activator (uPA). These enzymes degrade ECM components, triggering anoikis (detachment-induced apoptosis) and subsequent alveolar collapse [75,76]. MMP activity is tightly regulated by tissue inhibitors of metalloproteinases (TIMPs) to ensure balanced remodeling.

The driving force of this phase is mitochondrial-mediated apoptosis, regulated by the Bcl-2 family of proteins, which includes pro- and anti-apoptotic members [77]. Anti-apoptotic proteins (e.g., Bcl-2, Bcl-XL) maintain mitochondrial membrane integrity, while pro-apoptotic proteins (e.g., BAX, BAK, BID) promote membrane permeabilization [65]. Notably, the expression levels of Bcl-2 family expression are not influenced by STAT3 signaling but arise from the alleviation of repression imposed by STAT5 [77]. Apoptosis is initiated by the cleavage of BID into its truncated form (tBID), which activates BAX and BAK [62]. These proteins oligomerize, forming macropores in the mitochondrial membrane, leading to mitochondrial outer membrane permeabilization (MOMP) [78,79,80]. MOMP marks the irreversible commitment to cell death by releasing pro-apoptotic factors, including cytochrome C, SMAC, and OMI, which neutralize inhibitors like XIAP [78]. Cytochrome C binds apoptotic protease-activating factor 1 (APAF1), forming the apoptosome, which activates caspase-9 and subsequently executioner caspases-3 and -7, driving cellular disintegration [81]. During this stage, OSM binds its receptor (OSMR), further activating STAT3, illustrating the continued interplay of signaling pathways throughout involution [82,83]. This phase culminates in ECM remodeling, epithelial clearance, and adipocyte repopulation, which restore the gland to its pre-pregnancy architecture.

### 4.3. Clinical Implications of Postlactational Involution

Postlactational involution is a highly regulated process involving programmed cell death, ECM remodeling, and immune modulation. While critical for returning the mammary gland to its pre-pregnancy state, this process creates a microenvironment that may be exploited by tumor cells, particularly during the postpartum period. The postpartum period represents a vulnerable window of heightened breast cancer risk, linked to disruptions in postlactational involution. The combination of inflammation, ECM remodeling, and persistent epithelial structures can create a tumor-promoting microenvironment. Improved understanding of the molecular mechanisms underlying involution offers opportunities for targeted interventions to mitigate this risk.

## 5. Postlactational Involution and Breast Cancer Risk

The postpartum period is increasingly recognized as a window of heightened susceptibility to breast cancer. Breast cancer diagnosed within a decade of childbirth, referred to as postpartum breast cancer (PPBC), is more aggressive and exhibits a higher likelihood of metastasis compared to cancers in nulliparous women or those diagnosed outside the postpartum timeframe [84]. This phenomenon suggests that unique physiological changes during postlactational involution contribute to this increased risk.

### 5.1. Immune Microenvironment and Tumor Progression

Postlactational involution is strongly influenced by the immune system, which creates an environment that can be exploited by tumor cells. The process of involution shares several features with wound healing, including inflammation, proliferation, and tissue remodeling—factors known to promote tumorigenesis. These include an increased expression of chemokines, elevated levels of inflammatory mediators, such as COX-2 (cyclooxygenase-2), the deposition of fibrillar collagen, lymphangiogenesis, and immune cell infiltration [85,86]. A significant proportion of immune cells in the involuting mammary gland are M2 macrophages, which promote cell proliferation and tissue repair. These macrophages are notably more abundant in involuting lobules than in adjacent lactational lobules in human breast tissue [85,86]. The predominance of M2 macrophages contributes to an immunosuppressive microenvironment that facilitates tumor progression.

### 5.2. COX-2 and Semaphorin 7A (SEMA7A)

COX-2, a key enzyme in prostaglandin synthesis, plays a crucial role in the inflammatory processes of involution. Its expression is increased in mammary epithelial cells during postlactational involution, contributing to a tumor-promoting immune microenvironment. Prostaglandin E2 (PGE2), the predominant prostaglandin in cancer, supports tumorigenesis by affecting both tumor cells and the surrounding tumor microenvironment. Semaphorin 7A (SEMA7A), a membrane-anchored protein, facilitates cell attachment and spreading. Studies in preclinical models of PPBC showed that SEMA7A promoted multiple established drivers of PPBC progression, including a reciprocal relationship between COX-2 and SEMA7A. The knockdown of SEMA7A reduced collagen deposition and COX-2 expression, while COX-2 knockdown similarly downregulated SEMA7A [87]. This interplay indicates that COX-2 and SEMA7A work synergistically to establish a pro-tumorigenic environment during postlactational involution.

### 5.3. Lymphangiogenesis

Lymphangiogenesis, or the formation of new lymphatic vessels, introduces immunosuppressive elements into the tumor microenvironment. It suppresses cytotoxic T lymphocytes targeting the tumor and induces tolerance in naïve T cells [88,89]. PGE2 drives inflammatory lymphangiogenesis by stimulating inflammatory cells to release vascular endothelial growth factors C and D (VEGF-C, VEGF-D). The occurrence of lymphangiogenesis during postlactational involution aligns with increased lymph node involvement observed in individuals with PPBC compared to nulliparous patients of similar age [90,91]. Enhanced lymphatic networks may accelerate tumor cell dissemination, contributing to the metastatic potential of PPBC.

### 5.4. Fibrillar Collagen Deposition

Fibrillar collagen plays a critical role in breast cancer risk and progression. Mammographically dense breast tissue, strongly associated with increased cancer risk, correlates with higher collagen content [92,93]. During involution, the transient deposition of a pro-tumorigenic extracellular matrix-containing fibrillar collagen may have lasting effects on breast cancers developing in this period [94]. These changes highlight the long-term implications of the ECM remodeling that occurs during involution.

### 5.5. DNA Damage and Inflammation

The inflammatory microenvironment associated with involution promotes the production of reactive oxygen and nitrogen species (RONS) by immune cells, which, while aiding tissue remodeling, also induce DNA damage in epithelial cells [95]. This damage can lead to mutations that drive the initiation and progression of cancer. The combination of genetic alterations and a pro-tumorigenic microenvironment heightens the risk of malignant transformation during the postpartum period.

## 6. Postlactational Involution vs. Age-Related Lobular Involution

**Figure 2:**
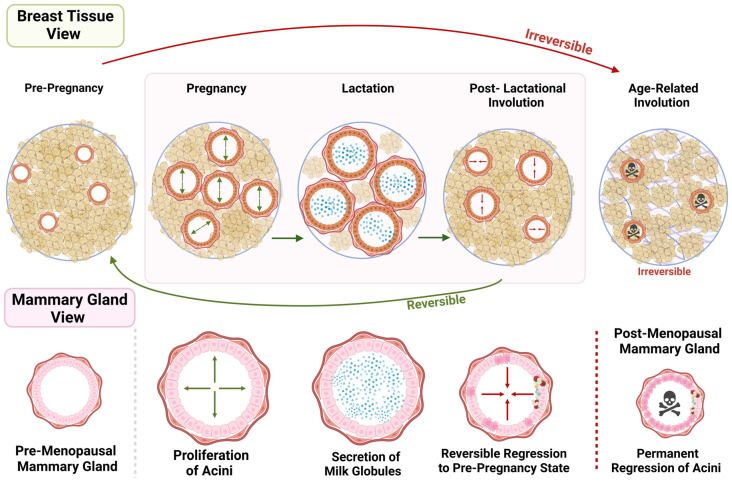
**Morphological timeline of mammary gland development and involution across life stages.** This figure illustrates the structural changes in mammary tissue during key life stages, from pre-pregnancy through lactation, postlactational involution, and aging. The Breast Tissue View (top panel) shows the overall organization of mammary tissue during different stages: Pre-Pregnancy: Sparse acini structures within adipose tissue. Pregnancy: Proliferation of acini as the gland prepares for lactation. Lactation: Acini are filled with milk globules to support breastfeeding. Postlactational Involution: Reversible regression occurs as secretory tissue remodels back to a pre-pregnancy state. Age-Related Involution: Irreversible loss of acini and replacement with adipose tissue, marked by senescent cell accumulation. The Mammary Gland View (bottom panel) depicts cellular-level changes: Pre-Menopausal Mammary Gland: Sparse epithelial-lined acini. Proliferation of Acini: Expansion during pregnancy. Secretion of Milk Globules: Characteristic of lactation. Reversible Regression: Postlactational involution restores tissue structure. Permanent Regression of Acini: During post-menopausal involution, glandular structures are permanently replaced by adipose tissue, reflecting irreversible aging-related changes. Arrows and Indicators: Green arrows: Reversible changes (e.g., pregnancy and lactation-induced growth and postlactational remodeling). Red arrows and skull icons: Irreversible changes associated with age-related involution and cellular senescence. This timeline underscores the distinct physiological processes and their reversibility or permanence, with implications for breast cancer risk at different life stages.

As women age, the mammary gland undergoes significant morphological and functional changes that affect breast cancer risk. A key physiological process necessary to maintain breast health in the aging mammary gland is age-related lobular involution (ARLI), characterized by the gradual regression of glandular structures, epithelial cell apoptosis, and the replacement of epithelial tissue with adipose tissue [96]. Both age-related lobular involution (ARLI) and postlactational involution involve mammary gland remodeling, but they differ significantly in their timing, mechanisms, and implications for breast cancer risk (Figure 2). Postlactational involution is a rapid process that occurs after weaning, during which the mammary gland returns to a non-lactating state through a tightly regulated sequence of apoptosis and tissue remodeling [58,97]. This process involves lysosomal-mediated cell death in the first phase and mitochondrial-mediated apoptosis in the second phase, with significant involvement of immune cells and extracellular matrix (ECM) remodeling [60,61].

In contrast, ARLI is a gradual and irreversible process associated with aging, characterized by the regression of lobular structures and prolonged tissue remodeling that occurs over decades [96]. While postlactational involution has been extensively studied in mouse models, ARLI presents a unique challenge because humans are the only known species whose breast tissue ages and persists after the childbearing years, making it impossible to experimentally model the molecular mechanisms of ARLI in animals [98]. ARLI begins during premenopause, accelerates during perimenopause, and culminates with the completion of menopause [99]. The implications of ARLI for breast cancer risk are significant. Complete ARLI, characterized by the comprehensive regression of lobular structures, appears protective against breast cancer by reducing the number of epithelial cells that could undergo malignant transformation. In contrast, incomplete ARLI, where tissue clearance is insufficient into the post-menopausal years, leaves behind a larger epithelial cell population within a potentially pro-tumorigenic microenvironment, which may contribute to an increased breast cancer risk [96,99]

Both involution processes involve apoptosis and remodeling, but postlactational involution is reversible and hormonally regulated, influenced by subsequent pregnancies, whereas ARLI is a progressive and irreversible process driven by intrinsic aging mechanisms. The immune responses also differ. Postlactational involution elicits a wound-healing-like immune response that, if dysregulated, can promote tumorigenesis [77]. ARLI, by contrast, is associated with chronic low-grade inflammation driven by the accumulation of senescent cells. These aging cells enter irreversible growth arrest but remain metabolically active, secreting inflammatory factors that alter the tissue environment. Together, these differences highlight how the intrinsic aging processes and incomplete tissue remodeling associated with ARLI create a microenvironment conducive to tumor development. Understanding these mechanisms is critical for developing strategies to mitigate breast cancer risk in aging populations.

## 7. Cellular Senescence in the Aging Mammary Gland

**Figure 3:**
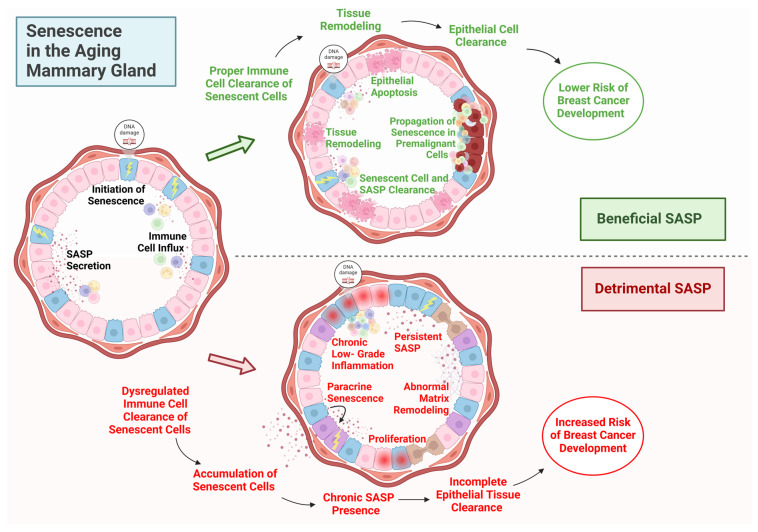
**The dual role of senescence and the senescence-associated secretory phenotype (SASP) in the aging mammary gland.** This conceptual figure illustrates the contrasting effects of senescence and SASP on breast tissue remodeling and breast cancer risk during aging, highlighting the distinction between beneficial and detrimental outcomes. Top Panel: Beneficial SASP: The initiation of senescence is triggered by DNA damage and stress signals, leading to SASP secretion and immune cell influx. The proper immune clearance of senescent cells and their SASP ensures effective tissue remodeling, epithelial apoptosis, and clearance of potentially pre-malignant cells. This process propagates senescence to other premalignant cells, contributing to tumor suppression and reduced cancer risk. Bottom Panel: Detrimental SASP: Dysregulated immune cell clearance results in the accumulation of senescent cells and persistent SASP secretion. Chronic SASP presence promotes low-grade inflammation, paracrine senescence, abnormal ECM remodeling, and epithelial proliferation. Incomplete epithelial tissue clearance and chronic SASP activity create a pro-tumorigenic environment, increasing the risk of breast cancer development. Arrows and Indicators: Green arrows: Beneficial effects of proper SASP regulation and senescence clearance, leading to reduced cancer risk. Red arrows: Pathological outcomes of dysregulated immune response and chronic SASP, contributing to a higher risk of malignancy. This schematic underscores the dual role of senescence in aging breast tissue and its impact on cancer risk, emphasizing the importance of balanced SASP regulation.

Cellular senescence is a state of irreversible cell cycle arrest induced by stressors such as telomere shortening, DNA damage, oxidative stress, and oncogene activation [92]. Despite their inability to divide, senescent cells remain metabolically active and secrete bioactive molecules collectively referred to as the SASP, which comprises pro-inflammatory cytokines, chemokines, growth factors, and matrix-remodeling enzymes, all of which exert profound effects on the surrounding tissue microenvironment [100] (Figure 3). Initiated in response to various stressors and damage [100], senescence exhibits a dual role: while senescent cells facilitate ARLI by promoting epithelial cell elimination during tissue remodeling, reducing the pool of epithelial cells susceptible to malignant transformation, the inflammatory secretome of senescent cells, known as the senescence-associated secretory phenotype (SASP), may impair the completion of ARLI and contribute to a pro-tumorigenic environment [100,101,102]. The interplay between ARLI and cellular senescence fundamentally shapes mammary tissue architecture and function throughout the aging process, balancing protective and pathological roles.

In the aging mammary gland, senescent cells accumulate due to an increased induction of senescence and decreased clearance by the immune system, a phenomenon known as immunosenescence [103,104]. Although cellular senescence initially acts as a tumor-suppressive mechanism by halting the proliferation of damaged cells, the chronic presence of senescent cells and their SASP can contribute to tissue dysfunction, chronic inflammation, and cancer development [93]. This dual role of senescence is particularly significant in the context of ARLI. Dysregulated senescence and incomplete ARLI may synergistically create a pro-tumorigenic microenvironment. SASP factors secreted by senescent cells may disrupt the normal progression of ARLI, affecting neighboring cells and the ECM, potentially delaying or preventing complete involution.

### 7.1. Senescence-Associated Secretory Phenotype (SASP) and Its Impact on ARLI

Senescent cells exert their most profound influence on tissue aging through the SASP, a potent network of inflammatory factors, growth modulators, and matrix-remodeling enzymes that dramatically reshape the local tissue environment and contribute to the progression of ARLI. The SASP is regulated through mechanisms both dependent on and independent of the DNA damage response [102,105]. These mechanisms operate at multiple levels, including gene activation, protein production, RNA stability, and secretion processes [106]. The composition and effects of the SASP vary significantly depending on the initial senescence-inducing trigger [107,108]. While the SASP is well-characterized in other aging tissues or in vitro models [109], its specific composition and dynamics during ARLI remain poorly understood. This knowledge gap is attributed to the complexity of mammary aging, which involves heterotypic signaling between multiple cell types, the challenge of obtaining sequential human breast tissue samples, and limitations in in vitro and animal models to replicate human breast tissue aging. Furthermore, significant inter-individual variability in senescence accumulation and ARLI progression, coupled with technological constraints, complicates the comprehensive study of these processes. The altered SASP from senescent cells can directly influence ARLI progression. Pro-inflammatory factors, growth factors, and matrix metalloproteinases (MMPs) disrupt normal tissue remodeling, potentially delaying or preventing complete involution. Such disruption may result in the persistence of epithelial cells and the creation of a microenvironment prone to malignant transformation.

#### 7.1.1. Pro-Inflammatory Factors

Dysregulated cellular senescence and aberrant ARLI establish a state of chronic inflammation in the aging mammary gland. Senescent cells secrete pro-inflammatory cytokines and chemokines, including interleukin-6 (IL-6), interleukin-8 (IL-8), and tumor necrosis factor-alpha (TNF-α), contributing to a sustained inflammatory milieu [110,111]. Chronic inflammation leads to increased oxidative stress through the production of reactive oxygen species (ROS) and reactive nitrogen species (RNS) [112], causing DNA damage and genomic instability in neighboring cells, significantly increasing the likelihood of malignant transformation [112].

The inflammatory environment may also alter the balance of signaling pathways involved in epithelial cell apoptosis and clearance during ARLI. Elevated IL-6 levels activate the STAT3 pathway, promoting epithelial cell survival and potentially hindering epithelial clearance, a hallmark of completed ARLI [113,114]. This persistence of epithelial cells increases the risk of malignancy. Additionally, chronic inflammation can induce epigenetic modifications in epithelial and stromal cells, activating oncogenic pathways or silencing tumor suppressor genes [115]. Persistent inflammation may also trigger fibrotic responses in the mammary stroma, impeding the replacement of epithelial tissue with adipose tissue, further disrupting normal tissue architecture and promoting tumor initiation [116].

#### 7.1.2. Growth Factors

The SASP includes growth factors, such as vascular endothelial growth factor (VEGF), epidermal growth factor (EGF), fibroblast growth factors (FGFs), and hepatocyte growth factor (HGF), which significantly impact the mammary microenvironment and potentially interfere with ARLI [108,117]. VEGF, for example, is primarily associated with angiogenesis but also promotes epithelial cell survival through anti-apoptotic pathways [118]. FGFs and EGFs stimulate epithelial cell proliferation and survival, which may counteract epithelial clearance during ARLI [119,120]. HGF, through interaction with the c-Met receptor, promotes epithelial cell survival, migration, and invasion, which may contribute to aggressive cancer phenotypes [121]. Growth factors also affect stromal cells, such as fibroblasts and endothelial cells, leading to tissue changes unfavorable to normal ARLI progression. For instance, VEGF-driven angiogenesis may sustain nutrient supply, promoting the survival of epithelial cells that should have been eliminated during ARLI [122].

#### 7.1.3. Matrix Remodeling Enzymes

Senescent cells secrete MMPs, such as MMP-3, MMP-9, and MMP-12, which degrade ECM components and promote aberrant remodeling [104,123]. Excessive ECM remodeling alters cell–matrix interactions, influencing epithelial cell behavior and survival during ARLI [124]. Additionally, excessive ECM stiffness, characterized by increased collagen deposition and crosslinking, is associated with heightened breast cancer risk by promoting malignant transformation through altered mechanotransduction signaling [125,126]. The remodeled ECM may also facilitate the formation of a pre-metastatic niche, potentially increasing the risk of tumor cell dissemination if cancer does develop [124]. Additionally, the altered ECM can affect the distribution and function of immune cells in the tissue, potentially compromising immune surveillance mechanisms that are crucial for eliminating pre-malignant cells [124].

#### 7.1.4. Alterations in Hormone Responsiveness

The interaction between senescence and ARLI could significantly alter the breast tissue’s response to hormonal stimuli, a critical factor in breast cancer development. Senescent cells may exhibit altered hormone receptor expression and increase local estrogen production through enhanced aromatase activity [127,128]. The persistence of hormone-responsive epithelial cells in incomplete ARLI, combined with elevated local estrogen levels, could lead to abnormal hormonal signaling, proliferation, and increased cancer risk [129]. The SASP also contains growth factors that could interact with hormone signaling pathways, creating a pro-proliferative environment in the incompletely involuted tissue [128]. This interaction could further increase the likelihood of malignant transformation.

#### 7.1.5. Immune Surveillance Modulation

The relationship between dysregulated senescence and delayed ARLI could also impact immune surveillance in breast tissue. While the SASP initially enhances immune recruitment, chronic inflammation shifts the microenvironment toward immunosuppression. Regulatory T cells (Tregs) and myeloid-derived suppressor cells (MDSCs) accumulate, inhibiting anti-tumor immune responses and facilitating tumor progression [130,131]. Over time, there is a shift toward an immunosuppressive microenvironment characterized by the accumulation of regulatory T cells (Tregs) and myeloid-derived suppressor cells (MDSCs) [101]. These immunosuppressive cells can inhibit the anti-tumor functions of cytotoxic T cells and natural killer (NK) cells, compromising the tissue’s ability to eliminate pre-malignant or malignant cells [132]. Chronic inflammation can also lead to T cell exhaustion, a state where T cells become less responsive to stimuli, further weakening immune surveillance [130]. Persistent senescent cells can also become immunogenic, continuously activating the immune system but eventually leading to immune tolerance. This is compounded by age-related immune decline, further compromising anti-tumor responses [133], continuously activating the immune system but potentially leading to a form of immune tolerance over time. This is exacerbated by the age-related decline in immune function, known as immunosenescence, which further compromises the ability to mount effective anti-tumor responses [130]. The dysregulation of local cytokine and chemokine gradients due to the SASP can disrupt normal immune cell trafficking and function, potentially leading to inappropriate immune responses or failure to respond to emerging threats [134]. This complex modulation of immune function creates an environment where pre-malignant cells are more likely to evade immune detection and elimination, significantly increasing the risk of tumor development and progression. Understanding these changes in immune surveillance in delayed ARLI could open avenues for potential therapeutic interventions, such as immunomodulatory treatments that could restore proper immune function and enhance the body’s natural ability to prevent cancer development.

### 7.2. Implications for Breast Cancer Development

Thus, dysregulated senescence and incomplete ARLI could synergistically contribute to a microenvironment conducive to breast cancer. The accumulation of senescent cells and their SASP interferes with ARLI, leading to epithelial persistence, chronic inflammation, ECM alterations, and immune dysfunction. These changes increase breast cancer risk through DNA damage, proliferation, and immunosuppression. Understanding these mechanisms highlights potential therapeutic strategies, including senolytics to clear senescent cells, senomorphics to modulate the SASP, and immunomodulatory treatments to restore immune function. Further research into these processes is essential for reducing breast cancer risk in aging populations.

## 8. Conclusions

The mammary gland is a uniquely dynamic organ, undergoing extensive morphological and functional changes throughout a woman’s life—from embryonic development to pregnancy, lactation, postlactational involution, and age-related lobular involution (ARLI). Each of these physiological processes is governed by tightly regulated signaling pathways and cellular interactions critical for normal breast development and function. However, disruptions or dysregulations in these processes can significantly influence breast cancer initiation, progression, and metastasis.

Embryonic mammary gland development establishes the structural foundation and cellular composition of the breast. Key signaling pathways, such as Wnt, FGF, SHH, Notch, EGFR, and BMP, are essential for proper development. Genetic mutations or environmental exposures that disrupt these pathways can predispose individuals to breast cancer later in life by affecting cell proliferation, differentiation, and survival mechanisms. A deeper understanding of these developmental pathways offers valuable insights into early detection and prevention strategies for breast cancer.

Postlactational involution represents another critical phase in mammary gland biology, wherein the gland remodels itself following lactation. This process, involving lysosomal-mediated cell death and mitochondrial-mediated apoptosis alongside extensive tissue remodeling, is tightly regulated. Dysregulation during postlactational involution can create a tumor-promoting microenvironment characterized by immune modulation, increased COX-2 and PGE2 production, lymphangiogenesis, and altered extracellular matrix (ECM) composition. These factors collectively contribute to the heightened aggressiveness and metastatic potential of PPBC. Further research into the molecular mechanisms linking postlactational involution to breast cancer risk is essential to develop targeted interventions. Such strategies could mitigate the increased postpartum risk, reducing the incidence and impact of PPBC. Although postlactational involution and ARLI share similarities in tissue remodeling and their influence on breast cancer risk, their mechanisms and timelines differ. Postlactational involution is an acute, hormonally regulated, and reversible process, whereas ARLI is a gradual and irreversible process driven by aging and chronic physiological changes. The chronic nature of ARLI and the interplay with senescence often lead to sustained pro-tumorigenic microenvironments, further compounding cancer risk in aging populations.

Emerging evidence reveals intriguing links between postlactational involution and cellular senescence in mammary tissue remodeling. During the irreversible phase of involution, senescent cells appear specifically in milk-producing luminal cells, marked by p16 expression and SASP production [135]. The experimental depletion of senescent cells using senolytics has been shown to impair involution, delaying tissue remodeling and adipocyte repopulation [135]. This connection between senescence and postlactational involution raises new questions about the complex cellular mechanisms underlying mammary tissue remodeling post-pregnancy and offers potential insights into the development of postpartum breast cancer.

Collectively, these physiological processes highlight the mammary gland’s remarkable dynamism and susceptibility to dysregulation at various life stages. Advancing our understanding of the molecular and cellular mechanisms underlying mammary gland development and involution has significant implications for breast cancer prevention and treatment. Targeting specific signaling pathways, modulating the tumor microenvironment, and enhancing immune function represent promising therapeutic strategies to mitigate breast cancer risk.

Future research should focus on elucidating the precise mechanisms linking developmental pathways to oncogenesis, identifying biomarkers for early detection, and developing approaches to normalize aberrant involution processes. Additionally, the exploration of senolytics and senomorphics to clear senescent cells or modulate the SASP may offer novel strategies for reducing breast cancer risk associated with aging. A detailed understanding of the molecular balance during these physiological processes will provide opportunities for interventions aimed at mitigating heightened breast cancer susceptibility during critical periods, such as postpartum and aging.

In conclusion, a comprehensive understanding of mammary gland development and involution—and their dysregulation—is essential for advancing breast cancer prevention, diagnosis, and treatment. Continued interdisciplinary research integrating developmental biology, oncology, immunology, and molecular genetics will be crucial for translating these insights into clinical applications. By unraveling the intricate mechanisms underlying breast cancer risk at different life stages, personalized strategies can be developed to address these unique vulnerabilities, ultimately reducing the burden of breast cancer.
